# Noninvasive ventilation for avoidance of reintubation in patients with various cough strength

**DOI:** 10.1186/s13054-016-1493-0

**Published:** 2016-10-07

**Authors:** Jun Duan, Xiaoli Han, Shicong Huang, Linfu Bai

**Affiliations:** Department of Respiratory Medicine, First Affiliated Hospital of Chongqing Medical University, Youyi Road 1, Yuzhong District, Chongqing, 400016 People’s Republic of China

**Keywords:** Noninvasive ventilation, Ventilator weaning, Cough strength, Reintubation

## Abstract

**Background:**

Reintubation is associated with high mortality. Identification of methods to avoid reintubation is needed. The aim of this study was to assess whether prophylactic noninvasive ventilation (NIV) would benefit patients with various cough strengths.

**Methods:**

We prospectively enrolled 356 patients who successfully passed a spontaneous breathing trial in a respiratory intensive care unit. Before extubation, cough peak flow was measured. After extubation, attending physicians determined whether the patients would receive prophylactic NIV or conventional oxygen treatment (control group). Patients were followed up to 90 days postextubation or death, whichever came first.

**Results:**

The median value of cough peak flow was 70 L/minute. Among the patients with cough peak flow ≤70 L/minute, 108 received NIV and 72 received conventional oxygen treatment. In this cohort, NIV reduced reintubation (9 % vs. 35 % at postextubation 72 h, *p* < 0.01; and 24 % vs. 49 % at postextubation 7 days, *p* < 0.01) and postextubation 90-day mortality (43 % vs. 61 %, *p* = 0.02) compared with the control group. Further, use of NIV was an independent protective factor for reintubation (OR = 0.19, *p* < 0.01 at 72 h postextubation; and OR = 0.33, *p* < 0.01 at 7 days postextubation) and for death at 90 days postextubation (OR = 0.40, *p* = 0.02). Among patients with cough peak flow >70 L/minute, 71 received NIV and 105 received conventional oxygen treatment. In this cohort, NIV did not reduce reintubation (6 % vs. 6 % at 72 h postextubation, *p* > 0.99; and 9 % vs. 9 % at 7 days postextubation, *p* > 0.99) or postextubation 90-day mortality (21 % vs. 15 %, *p* = 0.32) compared with the control group. Further, use of NIV was not associated with reintubation or postextubation 90-day mortality.

**Conclusion:**

In a planned extubated population, prophylactic NIV benefited patients with weak cough but possibly not in patients with strong cough.

## Background

Cough strength has been widely used to manage patients being removed from mechanical ventilation after a successful weaning test [1–6]. It is positively correlated with respiratory muscle strength [7]. Patients with weak cough are more likely to experience reintubation [1–6], and reintubation is associated with an eightfold increase in nosocomial pneumonia and a fivefold increase in death [8, 9]. Thus, it is necessary to identify effective methods to avoid reintubation.

Immediate use of prophylactic noninvasive ventilation (NIV) after extubation reduces reintubation in patients at high risk for extubation failure [10–13]. However, only one study enrolled patients with weak cough [13]. In that study, the authors enrolled only two patients with weak cough in the NIV group and three in the control group [13]. With such a small sample size, they failed to demonstrate the efficacy of prophylactic NIV in patients with weak cough. Further, to the best of our knowledge, no study to date has reported the efficacy of NIV in patients with strong cough. Therefore, the aim of this study was to determine whether prophylactic NIV would benefit patients with various cough strengths.

## Methods

The institutional review board of the First Affiliated Hospital of Chongqing Medical University approved this study. We prospectively enrolled patients who were scheduled for extubation after a successful spontaneous breathing trial (SBT) in a respiratory intensive care unit (ICU). We excluded patients younger than 18 years of age, with presence of a tracheostomy, or who refused to participate. Before enrollment, we obtained informed consent from the participants or their family members.

We managed the patients per our hospital’s protocols [6]. Every morning, we assessed each patient with regard to whether he or she met the criteria for removal of mechanical ventilation. We undertook an SBT if the following criteria were met: improvement or resolution of the underlying cause of acute respiratory failure, correction of arterial hypoxemia (ratio of partial pressure of arterial oxygen to fraction of inspired oxygen [PaO_2_/FiO_2_] ≥150, positive end-expiratory pressure ≤5 cmH_2_O), body temperature ≤38 °C, respiratory rate ≤30 breaths/minute, heart rate ≤120 beats/minute, and hemodynamic stability [14, 15]. The SBT was carried out in pressure support ventilation mode for 120 minutes. The support pressure was set at 6 cmH_2_O for an endotracheal tube inner diameter ≥7.5 mm and at 8 cmH_2_O for an endotracheal tube inner diameter <7.5 mm [14, 16]. We defined failure of the SBT as the presence one of the following criteria: respiratory rate ≥35 breaths/minute; frequency/tidal volume (rapid shallow breathing index) >105; peripheral oxygen saturation (SpO_2_) <90 % at FiO_2_ ≥ 0.5; heart rate ≥140 or ≤50 beats/minute; systolic blood pressure ≥180 or ≤90 mmHg; diminishing consciousness or diaphoresis; and clinical signs indicating respiratory muscle fatigue, labored breathing, or both. If no signs of SBT failure appeared after 120 minutes, the extubation was performed at the discretion of the attending physicians.

Before extubation, we recorded data for physiological variables, including Glasgow Coma Scale score. At the same time, from the nurse recording sheet, we recorded the suction frequency and volume of secretions preceding 24 h of extubation. We also measured the cough peak flow using a portable spirometer (Chestgraph HI-101; Chest M.I., Tokyo, Japan) [6]. Before measurement, we elevated the head of the bed to 30–45 degrees, cleared the airway secretions by suction, and oxygenated the patient with 100 % oxygen for 2 minutes. Next, we disconnected the ventilator, connected the spirometer to the endotracheal tube, and coached the patient to cough with as much effort as possible. We measured coughs three times, and the highest value was chosen. To avoid bias, the attending physicians were blind to the value of cough peak flow. Cough peak flow less than the median value was defined as weak cough.

After extubation, the attending physician determined whether the patient received prophylactic NIV or conventional oxygen treatment. We did not predefine the criteria for NIV. However, patients with weak hand-grip strength, high partial pressure of carbon dioxide in arterial blood, high Acute Physiology and Chronic Health Evaluation II score, low PaO_2_, and small volume of secretions were more likely to be ordered to receive prophylactic NIV. Prophylactic NIV (BiPAP Vision or V60; Philips Respironics, Monroeville, PA, USA) was immediately used after extubation. The face mask was the first choice. The appropriate size of the mask was selected according to the patient’s facial type. If a patient did not tolerate a face mask, a nasal mask was tried. The parameters of the ventilator were adjusted as follows. Expiratory positive airway pressure was set at 4–6 cmH_2_O. Inspiratory positive airway pressure was adjusted by increments of 1–2 cmH_2_O to obtain a tidal volume of around 8 ml/kg or to the maximum tolerated level for each patient. Usually, the inspiratory positive airway pressure was maintained at 12–20 cmH_2_O. FiO_2_ was set to maintain SpO_2_ at around 95 %. After 24 h, weaning from NIV was considered according to hospital protocol [17].

Reintubation was also determined by attending physicians on the basis of the following indicators (one major criterion or at least two minor criteria). The major criteria were (1) respiratory arrest, (2) loss of consciousness, (3) heart rate <50 beats/minute with loss of alertness, (4) development of conditions necessitating intubation to protect the airway (coma or seizure disorders) or copious tracheal secretions requiring management, and (5) hemodynamic instability without response to fluids and vasoactive drugs. The minor criteria were (1) respiratory rate >35 breaths/minute, (2) pH <7.35 for hypoxemic patients and <7.30 for hypercapnic patients, (3) PaO_2_ < 60 mmHg at FiO_2_ > 0.5 or supplemental oxygen flow >10 L/minute, (4) persistent tachycardia, and (5) persistent activation of accessory respiratory muscles.

We recorded whether the patient was reintubated within 72 h and within 7 days postextubation. We also recorded the duration of ICU stay, duration of hospital stay, duration of postextubation ICU stay, and duration of postextubation hospital stay when a patient was discharged from or died in the hospital. We followed the patient up to 90 days postextubation or death, whichever came first.

SPSS version 17.0 software (SPSS, Chicago, IL, USA) was used to analyze the data. Mean and SD values were used to report normally distributed continuous variables. The difference in two groups was analyzed using an unpaired Student’s *t* test. Median and interquartile range values were used to report non-normally distributed continuous variables. The difference between two groups was analyzed with the Mann–Whitney *U* test. For grouped data, the chi-square and/or Fisher’s exact test was used. The cumulative 90-day survival probability was analyzed by creating Kaplan-Meier curves, and the difference between two groups was analyzed by log-rank test. *p* < 0.05 was considered to signify statistical significance.

## Results

We enrolled 356 patients in this study between January 2011 and May 2016. The median value of cough peak flow was 70 L/minute. The proportions of patients who received NIV were 60 % (108 of 180 patients) among those with cough peak flow ≤70 L/minute and 40 % (71 of 176 patients) among those with cough peak flow >70 L/minute. The demographics of the patients are summarized in Table 1.

**Table 1:** Baseline values between groups

	Cough peak flow ≤70 L/minute			Cough peak flow >70 L/minute			
	NIV (*n* = 108)	Control (*n* = 72)	*p* Value^a^	NIV (*n* = 71)	Control (*n* = 105)	*p* Value^a^	*p* Value^b^
Age, years	73 ± 12	74 ± 13	0.68	67 ± 14	58 ± 19	<0.01^c^	<0.01^c^
Females/males, *n*	32/76	33/39	0.04^c^	12/59	27/78	0.20	<0.01^c^
Reason for intubation							
AECOPD	74	31	<0.01^c^	46	26	<0.01^c^	<0.01^c^
Pneumonia	26	32	0.01^c^	13	36	0.03^c^	0.42
ARDS	2	5	0.12	9	22	0.23	<0.01^c^
Asthma	2	0	0.52	1	5	0.40	0.17
Other	4	4	0.72	2	16	0.01^c^	0.04^c^
APACHE II score							
Upon admission	24 ± 6	23 ± 6	0.46	21 ± 6	19 ± 7	0.02^c^	<0.01^c^
At extubation	13 ± 3	13 ± 3	0.65	12 ± 3	11 ± 3	0.02^c^	<0.01^c^
Intubation period before extubation, days	8 ± 12	7 ± 5	0.44	7 ± 5	5 ± 4	<0.01^c^	0.01^c^
Cough peak flow, L/minute	48 ± 14	44 ± 15	0.07	98 ± 34	108 ± 29	0.04^c^	<0.01^c^
Hemoglobin, g/dl	10.8 ± 2.3	10.4 ± 2.2	0.26	11.3 ± 2.5	11.3 ± 2.4	0.97	0.01^c^
Secretions, ml/24 h	75 ± 45	85 ± 54	0.18	72 ± 49	77 ± 73	0.61	0.56
Suction frequency/24 h	11 ± 4	13 ± 4	<0.01^c^	12 ± 4	12 ± 4	0.71	0.96
GCS score	14.7 ± 1.2	14.2 ± 1.8	0.06	14.9 ± 0.1	14.9 ± 0.1	0.80	<0.01^c^
Physiological parameters at extubation							
pH	7.42 ± 0.05	7.43 ± 0.05	0.30	7.45 ± 0.05	7.46 ± 0.05	0.25	<0.01^c^
PaCO_2_, mmHg	51 ± 13	45 ± 13	<0.01^c^	48 ± 11	39 ± 10	<0.01^c^	<0.01^c^
PaO_2_/FiO_2_	222 ± 63	269 ± 86	<0.01^c^	225 ± 55	265 ± 94	<0.01^c^	0.33
Respiratory rate, breaths/minute	23 ± 5	23 ± 5	0.65	23 ± 6	22 ± 5	0.09	0.44
Rapid shallow breathing index	60 ± 27	66 ± 24	0.12	55 ± 21	48 ± 23	0.06	<0.01^c^
Heart rate, beats/minute	99 ± 16	93 ± 16	0.02^c^	100 ± 17	94 ± 15	0.01^c^	0.96
Mean arterial pressure, mmHg	93 ± 13	90 ± 12	0.18	94 ± 12	93 ± 12	0.53	0.40

*Abbreviations: APACHE II* Acute Physiology and Chronic Health Evaluation II, *NIV* Noninvasive ventilation, *AECOPD* Acute exacerbation of chronic obstructive pulmonary disease, *ARDS* Acute respiratory distress syndrome, *GCS* Glasgow Coma Scale, *PaCO*_*2*_ Partial pressure of carbon dioxide in arterial blood, *PaO*_*2*_ */FiO*_*2*_ Ratio of partial pressure of arterial oxygen to fraction of inspired oxygen

^a^Difference in NIV versus control

^b^Difference in weak versus strong cough

^c^ *p* < 0.05

**Fig. 1:**
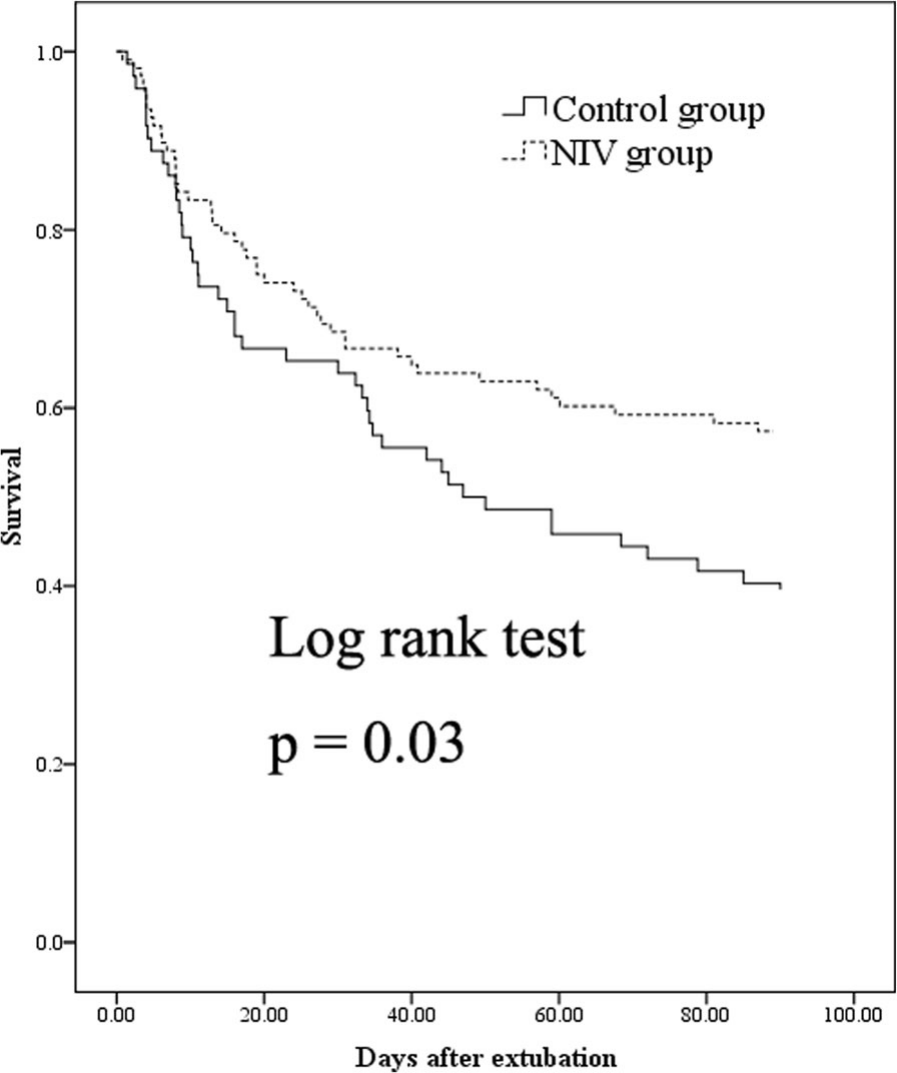
Cumulative 90-day survival in patients with cough peak flow ≤70 L/minute. *NIV* Noninvasive ventilation

In patients with cough peak flow ≤70 L/minute, NIV reduced reintubation at 72 h postextubation (10 of 108 [9 %] vs. 25 of 72 [35 %], *p* < 0.01) and 7 days postextubation (26 of 108 [24 %] vs. 35 of 72 [49 %], *p* < 0.01) compared with the control group (Table 2). It also reduced postextubation 90-day mortality (46 of 108 [43 %] vs. 44 of 72 [61 %], *p* = 0.02). In addition, NIV was a protective factor for reintubation at 72 h and 7 days postextubation (OR = 0.19, *p* < 0.01; OR = 0.33, *p* < 0.01) (Table 3). It also was a protective factor for death at 90 days postextubation (OR = 0.40, *p* = 0.02). Furthermore, patients in the NIV group had higher survival within 90 days postextubation (*p* = 0.03 by log-rank test) (Fig. 1).

**Table 2:** Outcomes between groups

	Cough peak flow ≤70 L/minute			Cough peak flow >70 L/minute			
	NIV (*n* = 108)	Control (*n* = 72)	*p* Value^a^	NIV (*n* = 71)	Control (*n* = 105)	*p* Value^a^	*p* Value^b^
Duration of ICU stay, days	13 (10–20)	15 (9–26)	0.34	12 (8–18)	9 (5–12)	<0.01^c^	<0.01^c^
Duration of hospital stay, days	23 (14–37)	26 (15–48)	0.16	19 (12–28)	17 (12–26)	0.40	<0.01^c^
Duration of postextubation ICU stay, days	6 (4–11)	7 (3–18)	0.52	5 (3–9)	3 (1–6)	<0.01^c^	<0.01^c^
Duration of postextubation hospital stay, days	13 (7–23)	14 (6–26)	0.78	10 (7–17)	10 (6–16)	0.54	<0.01^c^
Reintubation at 72 h postextubation	10 (9 %)	25 (35 %)	<0.01^c^	4 (6 %)	6 (6 %)	>0.99	<0.01^c^
Reintubation at 7 days postextubation	26 (24 %)	35 (49 %)	<0.01^c^	6 (9 %)	9 (9 %)	>0.99	<0.01^c^
Hospital mortality	36 (33 %)	33 (46 %)	0.12	8 (11 %)	14 (13 %)	0.82	<0.01^c^
Postextubation 90-day mortality	46 (43 %)	44 (61 %)	0.02^c^	15 (21 %)	16 (15 %)	0.32	<0.01^c^

*ICU* Intensive care unit, *NIV* Noninvasive ventilation

^a^Difference between NIV and control

^b^Difference between weak and strong cough

^c^ *p* < 0.05

**Table 3:** Multivariable analysis to identify independent risk factors for reintubation at 72 h and 7 days postextubation, and for death at 90 days postextubation

	Cough peak flow ≤70 L/minute		Cough peak flow >70 L/minute	
	OR (95 % CI)	*p* Value	OR (95 % CI)	*p* Value
Reintubation at 72 h postextubation				
Use of NIV	0.19 (0.09–0.43)	<0.01	–	N/A
APACHE II score at extubation	–	N/A	1.34 (1.10–1.63)	<0.01
Reintubation at 7 days postextubation				
Use of NIV	0.33 (0.16–0.66)	<0.01	–	N/A
Intubation period before extubation, days	1.07 (1.01–1.13)	0.02	–	N/A
Hemoglobin, g/dl	0.98 (0.96–0.99)	0.01	0.96 (0.94–0.99)	0.01
Cough peak flow, L/minute	0.97 (0.95–1.00)	0.04	–	N/A
APACHE II score at extubation	–	N/A	1.30 (1.08–1.56)	<0.01
Death at 90 days postextubation				
Use of NIV	0.40 (0.19–0.85)	0.02	–	N/A
Hemoglobin, g/dl	0.97 (0.95–0.99)	<0.01	0.97 (0.95–0.99)	<0.01
Cough peak flow, L/minute	0.96 (0.94–0.99)	<0.01	–	N/A
APACHE II score at extubation	1.18 (1.04–1.33)	0.01	1.37 (1.17–1.60)	<0.01

*Abbreviations: NIV* Noninvasive ventilation, *APACHE II* Acute Physiology and Chronic Health Evaluation II, *N/A* Not applicable

We entered age, sex, APACHE II score, intubation period, cough peak flow, hemoglobin, secretions, suction frequency, Glasgow Coma Scale score, heart rate, respiratory rate, rapid shallow breathing index, arterial blood gas tests, and use of NIV into multivariable analysis to identify independent risk factors for reintubation at 72 h and 7 days postextubation, and for death at 90 days postextubation

**Fig. 2:**
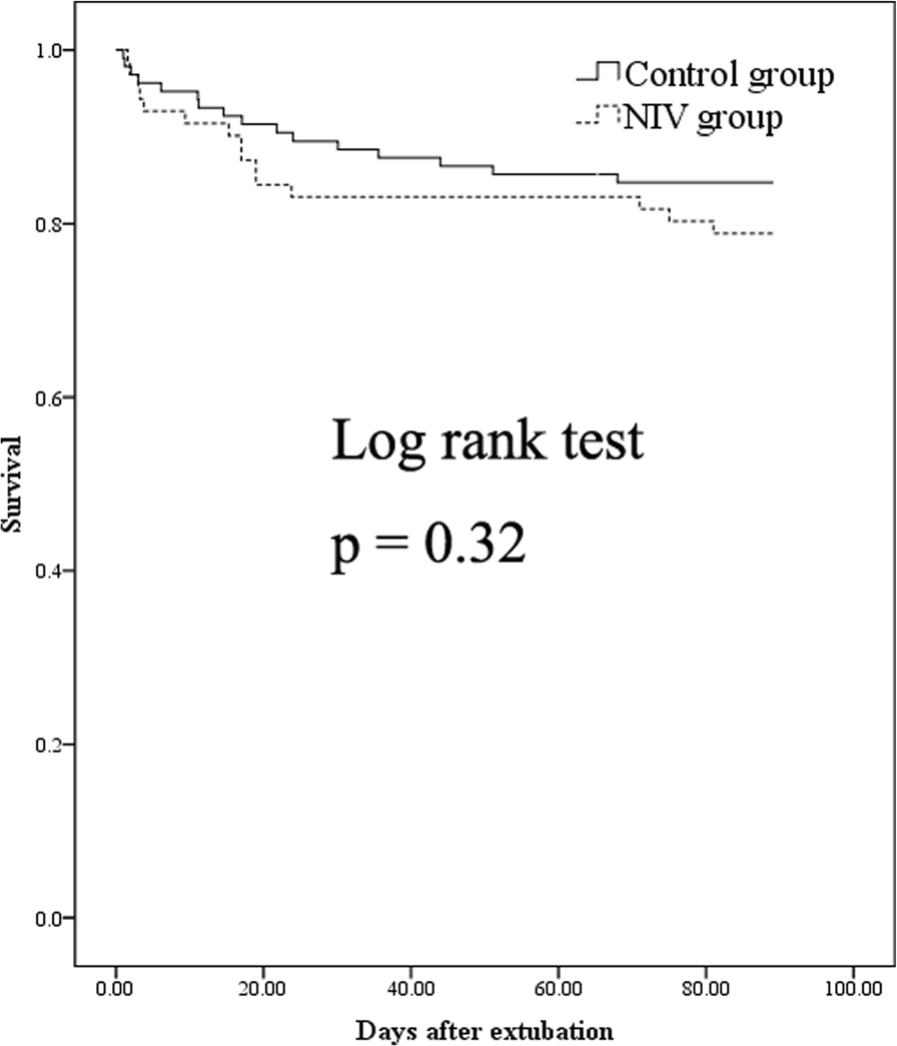
Cumulative 90-day survival in patients with cough peak flow >70 L/minute. *NIV* Noninvasive ventilation

In patients with cough peak flow >70 L/minute, NIV did not reduce reintubation (at 72 h postextubation: 4 of 71 [6 %] vs. 6 of 105 [6 %], *p* > 0.99; 7 days postextubation: 6 of 71 [9 %] vs. 9 of 105 [9 %], *p* > 0.99) or postextubation 90-day mortality (15 of 71 [21 %] vs. 16 of 105 [15 %], *p* = 0.32) compared with the control group (Table 2), nor was NIV associated with reintubation or postextubation 90-day mortality (Table 3). In addition, survival rates within 90 days postextubation were similar between the two groups (*p* = 0.32 by log-rank test) (Fig. 2).

The subgroup analysis of patients with chronic obstructive pulmonary disease (COPD) is summarized in Table 4. Prophylactic NIV was a protective factor for reintubation at 72 h postextubation (OR = 0.11, *p* < 0.01) and 7 days postextubation (OR = 0.27, *p* = 0.01) in patients with cough peak flow ≤70 L/minute. It was also a protective factor for death at postextubation 90 days in patients with weak cough (OR = 0.27, *p* = 0.01). However, prophylactic NIV was not associated with reintubation or postextubation 90-day mortality in patients with cough peak flow >70 L/minute.

**Table 4:** Multivariable analysis to identify independent risk factors for reintubation at 72 h and 7 days postextubation, and for death at 90 days postextubation, in patients with chronic obstructive pulmonary disease

	Cough peak flow ≤ 70 L/minute		Cough peak flow > 70 L/minute	
	OR (95 % CI)	*p* Value	OR (95 % CI)	*p* Value
Reintubation at 72 h postextubation				
Use of NIV	0.11 (0.03–0.38)	<0.01	–	N/A
Intubation period before extubation, days	1.10 (1.01–1.09)	0.03	–	N/A
Reintubation at 7 days postextubation				
Use of NIV	0.27 (0.10–0.77)	0.01	–	N/A
Cough peak flow, L/minute	0.95 (0.92–0.98)	<0.01	–	N/A
Death at 90 days postextubation				
Use of NIV	0.27 (0.10–0.74)	0.01	–	N/A
Hemoglobin, g/dl	0.98 (0.95–1.00)	0.02	0.97 (0.93–1.00)	0.05
Cough peak flow, L/minute	0.97 (0.94–1.00)	0.03	–	N/A
APACHE II score at extubation	1.21 (1.01–1.46)	0.04	1.46 (1.03–2.07)	0.04

*NIV* Noninvasive ventilation, *APACHE II* Acute Physiology and Chronic Health Evaluation II, N/A Not applicable

We entered age, sex, APACHE II score, intubation periods, cough peak flow, hemoglobin, secretions, suction frequency, Glasgow Coma Scale score, heart rate, respiratory rate, rapid shallow breathing index, arterial blood gas tests, and use of NIV into multivariable analysis to identify independent risk factors for reintubation at 72 h and 7 days postextubation, and for death at 90 days postextubation

## Discussion

To the best of our knowledge, this is the first study to report the efficacy of NIV in preventing reintubation in patients with weak cough strength (<70 L/minute). It also shows that when cough strength was >70 L/minute, reintubation was rare and NIV was not needed.

Respiratory muscle function is associated with ventilator weaning. Patients with greater respiratory muscle strength are more likely to wean from mechanical ventilation [18, 19], and respiratory muscle strength is positively correlated with cough peak flow [7]. Therefore, cough peak flow can serve as a predictor for successful weaning from mechanical ventilation. Several studies have reported that patients with lower cough peak flow had higher probability of reintubation [1–6]. However, how to reduce or avoid reintubation in this population is still unclear.

NIV reduces the work of breathing in patients with acute respiratory failure [20]. Given the advantages of NIV, it has been widely used in postextubation periods [10–13, 21, 22]. However, NIV benefited neither the entire population nor an unselected COPD population when it was used immediately after extubation [21, 22], but immediate use of NIV after extubation benefited patients at high risk for reintubation [10–13]. In our present study, we demonstrate that prophylactic NIV benefited patients with weak cough, including the COPD population, but that it did not benefit patients with strong cough with or without COPD. The results of this study may help practitioners to improve postextubation management.

To our knowledge, only one other study to date has been aimed at demonstrating the efficacy of prophylactic NIV in a high-risk population that included patients with weak cough [13]. In that study, the authors enrolled only five patients with weak cough. With such a small sample size, they failed to demonstrate the efficacy of prophylactic NIV in patients with weak cough. Further, they assessed the cough strength using Airway Care Score (a semiquantitative scale that includes six dimensions). However, cough peak flow is objective and has been widely used in cough strength assessment [1–6]. So, we selected a more objective and widely accepted measure to assess cough strength, which may be much easier to use in guiding clinical practitioners to manage ventilator weaning.

In patients with cough peak flow >70 L/minute, prophylactic NIV did not reduce reintubation or postextubation 90-day mortality. It indicated that patients with strong cough possibly received no benefit from prophylactic NIV. However, use of a high-flow nasal cannula benefited low-risk patients when it was used immediately after planned extubation [23]. Further, compared with NIV, it also showed benefits in patients with acute respiratory failure [24]. Thus, a high-flow nasal cannula was a good choice for postextubation management in patients with strong cough.

Our study may be limited by the methodology we used. It was an observational study, and the attending physicians determined whether the patients received NIV or conventional oxygen treatment. Patients with more serious illness were more likely to receive NIV. This led to unequal demographics between the NIV and control groups. However, we used multivariable logistic regression analysis and found that NIV was a protective factor for reintubation and for death at 90 days postextubation in patients with weak cough. Although a cohort study has less evidentiary strength than a randomized controlled study, our study with a large sample size shows the efficacy of prophylactic NIV in patients with weak cough strength.

## Conclusions

The median value of cough peak flow was 70 L/minute in a large planned extubation population. Prophylactic NIV benefited patients with weak cough with or without COPD, but not in patients with strong cough.

## Key messages

- The median value of cough peak flow in the planned extubated population was 70 L/minute.
- Immediate use of NIV after extubation reduced reintubation and postextubation 90-day mortality in patients with weak cough.
- However, prophylactic NIV may not have benefited patients with strong cough.

## Abbreviations

AECOPD: Acute exacerbation of chronic obstructive pulmonary disease
APACHE II: Acute Physiology and Chronic Health Evaluation II
ARDS: Acute respiratory distress syndrome
COPD: Chronic obstructive pulmonary disease
FiO_2_: Fraction of inspired oxygen
GCS: Glasgow Coma Scale
ICU: Intensive care unit
NIV: Noninvasive ventilation
PaCO_2_: Partial pressure of carbon dioxide in arterial blood
PaO_2_: Partial pressure of arterial oxygen
SBT: Spontaneous breathing trial
SpO_2_: Peripheral oxygen saturation
